# Transcatheter Versus Surgical Aortic Valve Replacement in Patients Aged 50 to 64 Years in the United States

**DOI:** 10.1016/j.jscai.2025.103991

**Published:** 2025-10-21

**Authors:** Mahmoud Ismayl, Hasaan Ahmed, Andrew M. Goldsweig, Sivakumar Sudhakaran, Kent Bailey, Juan Crestanello, Naveen Pereira, Mayra Guerrero

**Affiliations:** a Department of Cardiovascular Medicine, Mayo Clinic, Rochester, Minnesota; b Department of Cardiovascular Medicine, Baystate Medical Center, Springfield, Massachusetts; c Department of Quantitative Health Sciences, Mayo Clinic, Rochester, Minnesota; d Department of Cardiovascular Surgery, Mayo Clinic, Rochester, Minnesota

**Keywords:** aortic stenosis, outcomes, readmissions, surgical aortic valve replacement, transcatheter aortic valve replacement

## Abstract

**Background:**

Outcomes of transcatheter aortic valve replacement (TAVR) vs surgical aortic valve replacement (SAVR) in patients aged 50-64 years have not been evaluated in randomized clinical trials. Despite the lack of randomized data, these patients are often treated with TAVR.

**Methods:**

We queried the Nationwide Readmissions Database (2016-2021) to identify patients aged 50-64 years hospitalized for isolated aortic valve replacement (AVR). The contemporary use of TAVR and SAVR in patients aged 50-64 years was evaluated. In-hospital outcomes of TAVR vs SAVR were compared using propensity score matching. Readmissions were compared using the Cox proportional hazards regression model.

**Results:**

Of 75,413 weighted hospitalizations for isolated AVR in patients aged 50-64 years, 22,695 (30.1%) included TAVR, and 52,718 (69.9%) included SAVR. From 2016Q1 to 2021Q4, the proportion of AVR performed using TAVR increased from 12.6% to 41.4% in patients aged 50-64 years (p_trend_ < .001). TAVR, compared with SAVR, was associated with lower in-hospital mortality (1.0% vs 2.0%; *P* < .001), stroke (1.4% vs 2.8%; *P* < .001), acute kidney injury (9.8% vs 17.4%; *P* < .001), and major bleeding (1.0% vs 1.4%, *P* = .04) and with higher permanent pacemaker placement (4.7% vs 3.4%; *P* < .001), vascular complications (3.5% vs 1.6%; *P* < .001), and 180-day all-cause readmissions (14.4% vs 9.0%; *P* < .001). Length of stay was shorter (2 vs 6 days; *P* < .001) and nonhome discharges were lower (17.1% vs 54.6%; *P* < .001) with TAVR than those with SAVR.

**Conclusions:**

This nationwide observational analysis found that TAVR is increasingly performed among patients aged 50-64 years with lower in-hospital mortality and resource utilization but higher readmissions than SAVR.

## Introduction

Current US guidelines endorse differing recommendations for transcatheter aortic valve replacement (TAVR) and surgical aortic valve replacement (SAVR) in patients with severe aortic stenosis (AS) based on age.^1^ For patients between 65 and 80 years old among whom the use of a bioprosthetic valve is reasonable, shared decision making for TAVR vs SAVR selection is recommended (class 1 recommendations for both TAVR and SAVR).^1^ For patients >80 years old without anatomic contraindications, a bioprosthetic valve with TAVR is recommended (class 1 recommendation).^1^ For patients <65 years old, SAVR is recommended (class 1 recommendation) with a mechanical prosthetic valve for those <50 years old (class 2a recommendation) and either a mechanical or bioprosthetic valve for those between 50 and 65 years old (class 2a recommendation).^1^

Despite these guidelines, an increasing number of patients aged 50-64 years undergo TAVR, driven by randomized controlled trials supporting the expansion of TAVR to intermediate-risk and low-risk patient populations.^1^, ^2^, ^3^, ^4^, ^5^ The optimal choice for aortic valve replacement (AVR) among patients aged 50-64 years remains unclear, with limited evidence supporting the use of age-specific cutoffs for aortic valve interventions.^6^ While the PARTNER 3 and Evolut Low Risk trials found that TAVR is associated with superior and noninferior outcomes, respectively, compared with SAVR in low-risk individuals, its applicability to patients aged 50-64 years with AS remains limited, as the mean age of study participants was 73 and 74 years, respectively.^4^^,^^5^ Moreover, bicuspid aortic valve (BAV) was an exclusion criterion in both trials.^4^^,^^5^ Therefore, we queried the Nationwide Readmissions Database (NRD) to evaluate the relative utilization and comparative outcomes of TAVR vs SAVR among patients aged 50-64 years.

## Materials and methods

### Data source and ethics statement

Hospitalization data were abstracted from the NRD, which is part of the Healthcare Cost and Utilization Project (HCUP) family of databases sponsored by the Agency for Healthcare Research and Quality.^7^ The NRD is the largest publicly available, fully deidentified, all-payer inpatient health care readmission database in the United States. The NRD covers approximately 18 million unweighted hospitalizations each year with a diverse patient population, growing from 27 states in 2016 to 30 states in 2021 (Supplemental Table S1).^7^ When weighted, the NRD extrapolates to the national level, representing approximately 35 million hospitalizations each year. The unweighted sample represents approximately 50% of all US hospitalizations. Up to 40 discharge diagnoses and 25 procedure codes are collected for each patient using International Classification of Diseases, Tenth Revision (ICD-10) codes.^8^ The design and methodology of the NRD have been described previously.^9^, ^10^, ^11^ This study followed the STROBE (Strengthening the Reporting of Observational Studies in Epidemiology) reporting guideline (Supplemental Table S2)^12^ and was exempt from the requirements of the Mayo Clinic Institutional Review Board because the NRD is a fully deidentified, Health Insurance Portability and Accountability Act–compliant database that is publicly available from the HCUP website (www.hcup-us.ahrq.gov).

### Study population and patient selection

**Figure 1:**
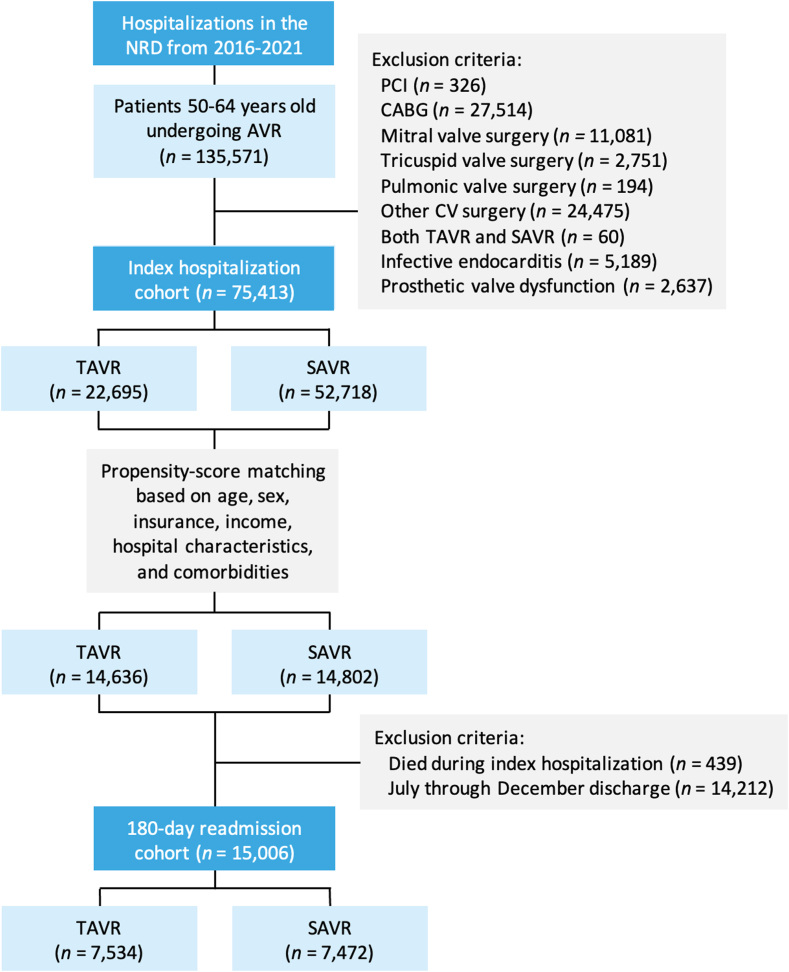
**Study flow diagram showing inclusion and exclusion criteria.** Hospitalization counts represent national level estimates. AVR, aortic valve replacement; CABG, coronary artery bypass grafting; CV, cardiovascular; NRD, Nationwide Readmissions Database; PCI, percutaneous coronary intervention; SAVR, surgical aortic valve replacement; TAVR, transcatheter aortic valve replacement.

We queried the NRD from January 2016 through December 2021 to identify hospitalizations in which patients aged 50-64 years underwent isolated AVR with TAVR (ICD-10, Procedure Coding System 02RF37H, 02RF38H, 02RF3JH, 02RF3KH, X2RF332, 02RF37Z, 02RF38Z, 02RF3JZ, and 02RF3KZ in any procedural field) or SAVR (ICD-10, Procedure Coding System 02RF07Z, 02RF08Z, 02RF0JZ, 02RF0KZ, and X2RF032 in any procedural field). We excluded hospitalizations in which patients were aged <50 years or ≥65 years as well as those with concomitant percutaneous coronary intervention, concomitant surgeries (coronary artery bypass grafting, mitral/tricuspid/pulmonic valve surgeries, aortic surgeries, and other cardiovascular surgeries), infective endocarditis, or prosthetic valve dysfunction (Figure 1). A complete list of ICD-10 diagnosis and procedure codes used in this study is presented in Supplemental Table S3.

When evaluating 180-day readmissions, we excluded hospitalizations in which the patient died during the index hospitalization as well as any discharge that occurred after June 30 of each calendar year because the NRD follows up patients within a single calendar year and does not capture readmissions across calendar years (Figure 1). Therefore, to enable a full 180-day follow-up for all discharges, we used data only from January 1 to June 30 of each year for the analysis on 180-day readmissions. In patients who had multiple 180-day readmissions, only the first readmission was included in the analysis. The NRD does not provide data on out-of-hospital deaths, and therefore, patients who died at home within 180 days were counted as patients without a readmission within 180 days.

### Study outcomes

The temporal trends in isolated TAVR and SAVR use in patients aged 50-64 years were reported. The primary outcome was in-hospital all-cause mortality. Secondary outcomes included in-hospital complications of stroke, heart block, permanent pacemaker (PPM) placement, acute kidney injury (AKI), major bleeding, blood transfusion, and vascular complications (defined as a composite of arteriovenous fistula, aneurysm, hematoma, retroperitoneal bleeding, and venous thromboembolism), as well as hospital length of stay (LOS), total hospital costs (inflation adjusted to 2021 US dollars),^13^ and discharge disposition. Charge-to-cost ratio files were used to convert charges to costs at the individual hospital level. All-cause, heart failure, and stroke readmissions at 90 and 180 days were also evaluated.

### Statistical analysis

Normality of continuous data was assessed using the Shapiro–Wilk or Kolmogorov–Smirnov test, as appropriate. Continuous variables were reported as mean ± SD for normal distributions or median and IQR for skewed distributions. Categorical variables were reported as percentages. Comparisons were made between the 2 groups using the Student *t* test or Mann–Whitney *U* test for continuous variables and the Pearson χ^2^ or Fisher exact test for categorical variables, as appropriate.

Temporal changes in hospitalizations for TAVR and SAVR were analyzed using linear regression. Propensity score matching methodology was performed to match patients aged 50-64 years who underwent isolated TAVR to those who underwent isolated SAVR, respectively, in a 1:1 ratio. Each case was propensity score matched to a control using nearest neighbor technique with a caliper width of 0.2 (Supplemental Figures S1-S4). The propensity score was calculated from the following variables: age, sex, primary payer, median income quartile by ZIP Code, hospital location (urban/rural) and teaching status, number of hospital beds, admission type (elective/nonelective) and day (weekend/weekday), BAV, Elixhauser and Charlson comorbidity index scores, and relevant comorbidities (Supplemental Table S4) using R’s MatchIt package.^14^ Adjustment variables were selected a priori on the basis of their clinical significance and on their likely influence on in-hospital outcomes and readmissions. Logistic regression analysis was performed to estimate odds ratios with 95% CIs.

The probabilities of 90- and 180-day readmission, stratified on the basis of TAVR vs SAVR, were graphically displayed using the Kaplan–Meier method and compared using the log-rank test. Cox proportional hazards regression analysis was performed to estimate hazard ratios (HRs) with their corresponding 95% CIs. The assumptions of Cox proportional hazards regression were graphically assessed using log-log plots and tested based on Schoenfeld residuals.

Complete data were available for all variables except for primary payer (missing, 0.1%), median household income quartile by ZIP code (missing, 1.4%), and type of admission (missing, 0.1%). As the overall missing values were minimal (<1.5%) and limited to only 3 of the potential confounding variables, they were assumed to be missing at random, and the level of bias was likely small. Missing values were handled with listwise deletion and were not included in the regression analysis.

For all statistical analyses, a 2-tailed *P* < .05 was considered statistically significant. Given the large sample size, not all statistically significant *P* values represent clinically significant differences and therefore require careful interpretation. All statistical analyses were performed using Stata version 17 (StataCorp) software and R software for Statistical Computing, version 4.3 (R Foundation for Statistical Computing), accounting for the NRD sampling design, and were weighted using sampling weights provided with the NRD to estimate national-level effects per HCUP-NRD recommendations similar to previous studies.^7^^,^^15^, ^16^, ^17^, ^18^

## Results

### Patient and hospital characteristics

From January 2016 through December 2021, 75,413 hospitalizations in the NRD met inclusion criteria, of which 22,695 (30.1%) included TAVR and 52,718 (69.9%) included SAVR (Figure 1). Patients who underwent TAVR were older, more likely to be female and to have Medicare insurance, and less likely to have a BAV compared with patients who underwent SAVR. TAVR-treated patients had higher Elixhauser and Charlson comorbidity index scores, driven primarily by higher rates of diabetes, hypertension, dyslipidemia, nicotine/tobacco use, obesity, coronary artery disease, congestive heart failure, renal failure requiring dialysis, liver disease, chronic pulmonary disease, obstructive sleep apnea, and cancer. Furthermore, TAVR-treated patients were more likely to have a history of myocardial infarction, stroke/transient ischemic attack, cardiac arrest, percutaneous coronary intervention, coronary artery bypass grafting, or preexisting implantable cardioverter-defibrillator or PPM. Baseline characteristics of the unmatched and matched cohorts, stratified by TAVR vs SAVR procedures, are shown in Table 1.

**Table 1:** Baseline characteristics stratified by TAVR vs SAVR.

	Unmatched			Propensity score matched^a^		
	TAVR (n = 22,695)	SAVR (n = 52,718)	*P*	TAVR (n = 14,636)	SAVR (n = 14,802)	*P*
Demographic characteristics						
Age, y	61 (58-63)	59 (55-62)	<.001	60 (57-63)	60 (57-63)	.65
Biological sex						
Male	62.5	67.9	<.001	63.7	64.0	.70
Female	37.5	32.1		36.3	36.0	
Primary payer						
Medicare	34.9	13.0	<.001	23.2	24.7	.38
Medicaid	13.3	11.6		14.4	14.1	
Private insurance	45.6	68.8		55.6	54.5	
Self-pay	2.0	2.8		2.5	2.5	
Other	4.2	3.8		4.3	4.2	
Income quartile^b^						
I	26.7	23.0	<.001	25.2	26.2	.51
II	29.2	28.8		29.3	29.2	
III	25.3	26.8		25.6	25.6	
IV	18.8	21.4		19.9	19.0	
Hospital characteristics						
Location/teaching status						
Rural	8.5	12.2	<.001	9.4	9.3	.47
Urban nonteaching	90.4	86.4		89.4	89.2	
Urban teaching	1.1	1.4		1.2	1.5	
Bed size^c^						
Small	5.0	7.7	<.001	5.6	5.5	.93
Medium	18.6	22.7		19.9	19.5	
Large	76.4	69.6		74.5	75.0	
Elective admission	77.4	78.1	.24	75.8	75.6	.75
Weekend admission	4.7	4.8	.45	4.9	5.5	.10
Clinical characteristics						
Bicuspid aortic valve	8.8	28.1	<.001	12.6	11.2	.06
Elixhauser comorbidity index	6 (4-7)	5 (4-6)	<.001	5 (4-7)	5 (4-7)	.10
Charlson comorbidity index	3 (2-5)	1 (1-2)	<.001	2 (1-3)	2 (1-4)	.01
0	6.0	24.5	<.001	9.0	8.9	.15
1	16.4	30.2		23.2	22.6	
2	19.7	20.9		24.5	23.3	
≥3	57.9	24.4		43.3	45.2	
Diabetes mellitus	45.2	28.1	<.001	37.4	38.8	.12
Hypertension	85.1	76.3	<.001	82.0	81.6	.62
Dyslipidemia	65.8	59.4	<.001	63.6	62.8	.34
Nicotine/tobacco use	46.4	44.4	.001	45.8	46.8	.27
Alcohol abuse	5.4	4.9	.06	5.8	5.7	.76
Drug abuse	3.0	3.6	<.001	3.4	3.3	.75
Obesity	36.2	33.6	<.001	36.8	37.2	.70
Coronary artery disease	59.2	34.4	<.001	48.0	49.6	.09
Peripheral vascular disease	19.5	19.1	.42	17.6	17.2	.56
Atrial fibrillation/flutter	23.7	34.6	<.001	26.7	26.7	.93
Congestive heart failure	74.7	39.1	<.001	64.9	65.9	.37
Renal failure	31.6	12.2	<.001	20.7	22.0	.09
Dialysis dependent	10.0	1.6	<.001	3.4	4.2	.01
Liver disease	10.9	5.1	<.001	9.0	9.2	.72
Chronic pulmonary disease	33.4	21.8	<.001	28.9	30.4	.08
Obstructive sleep apnea	23.5	18.3	<.001	21.3	21.4	.92
Coagulopathy	13.0	30.8	<.001	16.7	16.0	.37
Cancer	4.3	1.5	<.001	2.7	2.9	.50
Malnutrition	1.8	1.6	.26	2.0	1.9	.72
Dementia	0.6	0.2	<.001	0.4	0.4	.98
Depression	13.1	12.1	.02	13.1	13.2	.81
History						
Myocardial infarction	12.1	4.9	<.001	8.1	9.3	.01
Stroke/TIA	8.8	5.2	<.001	6.7	7.1	.38
Cardiac arrest	0.8	0.4	<.001	0.5	0.5	.61
PCI	16.5	4.9	<.001	9.1	10.4	.02
CABG	11.8	2.4	<.001	4.4	5.8	<.001
ICD	3.2	0.9	<.001	1.7	1.9	.37
PPM	3.3	1.5	<.001	2.4	2.7	.24

Data presented as median (IQR) or %. Two authors (M.I. and H.A.) independently verified the International Classification of Diseases, Tenth Revision (ICD-10) codes that corresponded to each comorbidity (Supplemental Table S3), and any disagreements in inclusion or exclusion of ICD-10 codes were discussed with a third author (A.M.G).

CABG, coronary artery bypass grafting; ICD, implantable cardioverter-defibrillator; PCI, percutaneous coronary intervention; PPM, permanent pacemaker; SAVR, surgical aortic valve replacement; TAVR, transcatheter aortic valve replacement; TIA, transient ischemic attack.

a Propensity score matching was performed using age, sex, primary payer, median income quartile, hospital location (urban/rural) and teaching status, number of hospital beds, admission type (elective/nonelective) and day (weekend/weekday), bicuspid aortic valve, Elixhauser and Charlson comorbidity index scores, and relevant comorbidities (Supplemental Table S4).

b Estimated median household incomes are specific to ZIP code, updated annually, and classified into 4 quartiles indicating the poorest to wealthiest populations.

c Bed size categories are based on inpatient number of beds and are specific to the hospital’s location and teaching status. A more detailed explanation of all variables in the NRD, including the specific dollar amounts in each category of median household income and the number of hospital beds in each category, is available online (https://hcup-us.ahrq.gov/db/nation/nrd/nrddde.jsp).

### Trend analysis

**Figure 2:**
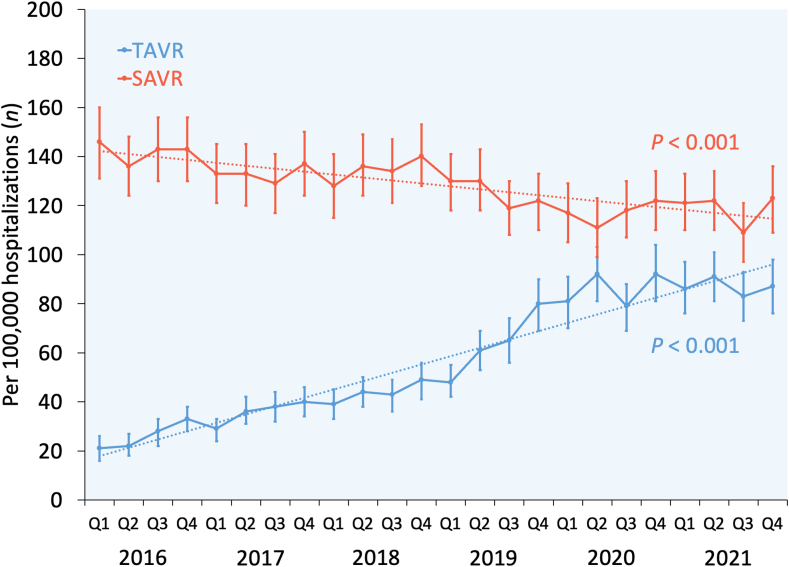
**Year-over-year trend in the use of TAVR vs SAVR among patients aged 50-64 years in the United States from 2016 through 2021.** Error bars represent 95% CIs. Dotted lines represent linear trends. SAVR, surgical aortic valve replacement; TAVR, transcatheter aortic valve replacement.

From 2016Q1 through 2021Q4, the overall use of AVR increased from 167 to 210 per 100,000 hospitalizations in patients aged 50-64 years (*P*_trend_ < .001). While the use of TAVR increased from 21 to 87, the use of SAVR decreased from 146 to 123 per 100,000 hospitalizations in patients aged 50-64 years (both *P*_trend_ < .001). By the last quarter of 2021, the proportion of AVR performed using TAVR in patients aged 50-64 years reached 41.4%. Annual trends for TAVR vs SAVR in patients aged 50-64 years are shown in Figure 2.

### In-hospital outcomes

The estimated overall in-hospital all-cause mortality rate was 1.5% (95% CI, 1.3%-1.7%). After propensity score matching 29,438 patients (14,636 TAVR and 14,802 SAVR), those who underwent TAVR had lower rates of in-hospital mortality (1.0% vs 2.0%; *P* < .001), stroke, AKI, major bleeding, and blood transfusion, and higher rates of heart block, PPM placement, and vascular complications compared with patients who underwent SAVR (Table 2).

**Table 2:** In-hospital outcomes stratified by TAVR vs SAVR.

	Unmatched				Propensity score matched^a^			
	TAVR (n = 22,695)	SAVR (n = 52,718)	Odds ratio (95% CI)	*P*	TAVR (n = 14,636)	SAVR (n = 14,802)	Odds ratio (95% CI)	*P*
Mortality	1.0	1.2	0.82 (0.65-1.05)	.12	1.0	2.0	0.50 (0.37-0.67)	<.001
Stroke	1.6	2.1	0.76 (0.63-0.91)	.003	1.4	2.8	0.48 (0.37-0.61)	<.001
Heart block	25.4	13.3	2.22 (2.08-2.38)	<.001	25.0	12.6	2.31 (2.10-2.55)	<.001
PPM placement	4.3	3.8	1.11 (0.99-1.25)	.06	4.7	3.4	1.40 (1.18-1.66)	<.001
Acute kidney injury	9.7	13.8	0.67 (0.62-0.74)	<.001	9.8	17.4	0.51 (0.46-0.57)	<.001
Major bleeding	1.0	1.7	0.62 (0.48-0.78)	<.001	1.0	1.4	0.72 (0.51-0.94)	.04
Blood transfusion	4.9	12.7	0.35 (0.31-0.39)	<.001	4.3	14.0	0.28 (0.24-0.32)	<.001
Vascular complications	3.4	1.6	2.20 (1.89-2.56)	<.001	3.5	1.6	2.25 (1.77-2.86)	<.001

Data presented as %.

PPM, permanent pacemaker; SAVR, surgical aortic valve replacement; TAVR, transcatheter aortic valve replacement.

a Propensity score matching was performed using age, sex, primary payer, median income quartile, hospital location (urban/rural) and teaching status, number of hospital beds, admission type (elective/nonelective) and day (weekend/weekday), bicuspid aortic valve, Elixhauser and Charlson comorbidity index scores, and relevant comorbidities (Supplemental Table S4).

Patients treated with TAVR had a shorter median hospital LOS (2 vs 6 days; *P* < .001) and similar median total costs ($54,829 vs $54,653; *P* = .99) compared with those treated with SAVR (Table 3). For hospitalizations in which the patient was discharged alive, TAVR-treated patients were discharged at greater rates to home without services (82.9% vs 45.4%; *P* < .001) as opposed to home health care, skilled nursing, or intermediate care facilities compared with SAVR-treated patients (Table 3).

**Table 3:** Discharge disposition and resource utilization stratified by TAVR vs SAVR.

	Unmatched			Propensity score matched^a^		
	TAVR (n = 22,695)	SAVR (n = 52,718)	*P*	TAVR (n = 14,636)	SAVR (n = 14,802)	*P*
Discharge disposition						
Routine	81.7	48.7	<.001	82.9	45.4	<.001
Transfer to short-term hospital	0.2	0.3		0.3	0.4	
Transfer to SNF or ICF	4.7	6.4		4.3	9.1	
Home health care	13.4	44.6		12.5	45.1	
Resource utilization						
LOS, d	2 (1-5)	6 (4-9)	<.001	2 (1-5)	6 (5-10)	<.001
Hospital cost, $	55,197 (38,233-88,486)	50,771 (35,240-84,686)	<.001	54,829 (38,156-88,089)	54,653 (36,777-94,342)	.99

Data presented as median (IQR) or %.

ICF, intermediate care facility; LOS, length of stay; SAVR, surgical aortic valve replacement; SNF, skilled nursing facility; TAVR, transcatheter aortic valve replacement.

a Propensity score matching was performed using age, sex, primary payer, median income quartile, hospital location (urban/rural) and teaching status, number of hospital beds, admission type (elective/nonelective) and day (weekend/weekday), bicuspid aortic valve, Elixhauser and Charlson comorbidity index scores, and relevant comorbidities (Supplemental Table S4).

### The 90- and 180-day readmission rates

The estimated overall 90-day all-cause readmission rate was 8.6% (95% CI, 8.1%-9.0%) and 180-day all-cause readmission rate was 11.6% (95% CI, 11.1%-12.1%). The most common cause for readmission was heart failure at both 90 days (1.3%; 95% CI, 1.1%-1.4%) and 180 days (1.9%; 95% CI, 1.7%-2.1%).

**Figure 3:**
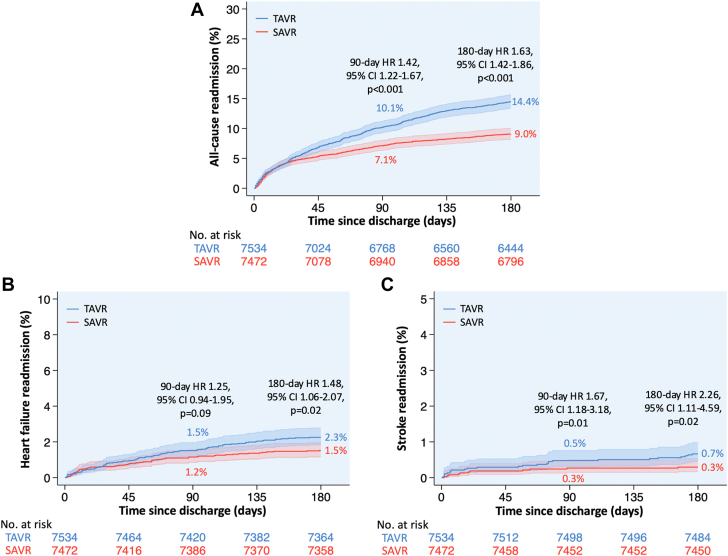
**Kaplan-Meier curves of all-cause (A), heart failure (B), and stroke (C) readmissions at 90 and 180 days following TAVR vs SAVR in propensity score–matched∗ patients aged 50-64 years.** ∗Propensity score matching was performed using age, sex, primary payer, median income quartile, hospital location (urban/rural) and teaching status, number of hospital beds, admission type (elective/nonelective) and day (weekend/weekday), bicuspid aortic valve, Elixhauser and Charlson comorbidity index scores, and relevant comorbidities (Supplemental Table S4). HR, hazard ratio; SAVR, surgical aortic valve replacement; TAVR, transcatheter aortic valve replacement.

After propensity score matching 15,006 patients (7534 TAVR and 7472 SAVR), TAVR was associated with higher 90-day all-cause and stroke readmissions and 180-day all-cause, heart failure, and stroke readmissions compared with SAVR. Readmissions stratified by TAVR vs SAVR are presented in Figure 3 and Table 4.

**Table 4:** Readmissions stratified by TAVR vs SAVR.

	Unmatched				Propensity score matched^a^			
	TAVR (n = 10,583)	SAVR (n = 26,072)	Hazard ratio (95% CI)	*P*	TAVR (n = 7534)	SAVR (n = 7472)	Hazard ratio (95% CI)	*P*
90 d readmission								
All-cause readmission	11.5	5.7	2.06 (1.85-2.29)	<.001	10.1	7.1	1.42 (1.22-1.67)	<.001
Heart failure readmission	1.9	0.7	2.67 (2.02-3.53)	<.001	1.5	1.2	1.25 (0.94-1.95)	.09
Stroke readmission	0.6	0.2	2.79 (1.62-4.82)	<.001	0.5	0.3	1.67 (1.18-3.18)	.01
180 d readmission								
All-cause readmission	16.0	7.1	2.32 (2.11-2.55)	<.001	14.4	9.0	1.63 (1.42-1.86)	<.001
Heart failure readmission	2.8	0.9	3.04 (2.39-3.87)	<.001	2.3	1.5	1.48 (1.06-2.07)	.02
Stroke readmission	0.7	0.2	2.96 (1.83-4.79)	<.001	0.7	0.3	2.26 (1.11-4.59)	.02

Data presented as % according to Kaplan–Meier estimate. The *P* values are based on log-rank test.

SAVR, surgical aortic valve replacement; TAVR, transcatheter aortic valve replacement.

a Propensity score matching was performed using age, sex, primary payer, median income quartile, hospital location (urban/rural) and teaching status, number of hospital beds, admission type (elective/nonelective) and day (weekend/weekday), bicuspid aortic valve, Elixhauser and Charlson comorbidity index scores, and relevant comorbidities (Supplemental Table S4).

## Discussion

This analysis of patients aged 50-64 years undergoing AVR using the large, nationally representative NRD yielded several novel findings (Central Illustration): (1) the use of TAVR increased while the use of SAVR decreased from 2016 through 2021; (2) compared with SAVR, TAVR was associated with lower odds of in-hospital mortality, stroke, AKI, major bleeding, and blood transfusion, and higher odds of heart block, PPM placement, and vascular complications; (3) LOS was shorter and nonhome discharges were lower with TAVR than those with SAVR; and (4) 90-day all-cause and stroke readmissions and 180-day all-cause, heart failure, and stroke readmissions were higher with TAVR than those with SAVR.

**Central Illustration:**
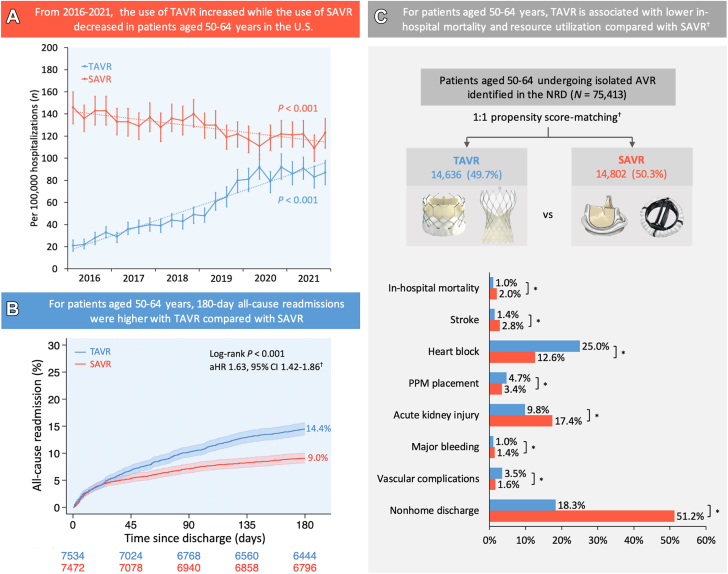
TAVR vs SAVR in propensity score–matched patients aged 50-64 years in the United States. (**A**) Year-over-year trend of TAVR vs SAVR in patients aged 50-64 years. (**B**) Comparison of 180-day all-cause readmissions following TAVR vs SAVR in patients aged 50-64 years. (**C**) Comparison of TAVR vs SAVR in-hospital outcomes in patients aged 50-64 years. ∗*P* < .05. ^†^Propensity score matching was performed using age, sex, primary payer, median income quartile, hospital location (urban/rural) and teaching status, number of hospital beds, admission type (elective/nonelective) and day (weekend/weekday), bicuspid aortic valve, Elixhauser and Charlson comorbidity index scores, and relevant comorbidities (Supplemental Table S4). aHR, adjusted hazard ratio; AVR, aortic valve replacement; NRD, Nationwide Readmissions Database; PPM, permanent pacemaker; SAVR, surgical aortic valve replacement; TAVR, transcatheter aortic valve replacement.

The approval of TAVR progressed in a stepwise process across surgical risk groups. In 2011, the US Food and Drug Administration approved TAVR for patients with severe symptomatic AS who were deemed inoperable, based on the PARTNER 1B trial, which noted a survival benefit with TAVR over standard therapy.^19^ In 2012, approval expanded to high-risk patients following the PARTNER 1A trial, which demonstrated noninferiority of TAVR compared with SAVR.^20^ The CoreValve High Risk trial further supported this in 2014 by showing superior 1-year survival with TAVR, compared with SAVR, among high-risk patients.^21^ In 2016, TAVR was approved for intermediate-risk patients after both the PARTNER 2A and SURTAVI trials established noninferiority over SAVR.^2^^,^^3^ Finally, in 2019, TAVR received approval for low-risk patients based on the PARTNER 3 and Evolut Low Risk trials, which demonstrated superior and noninferior outcomes, respectively, compared with SAVR.^4^^,^^5^

The decision to proceed with TAVR vs SAVR in patients aged 50-64 years is often challenging, as providers and patients must consider lifetime management due to the eventual structural deterioration of bioprosthetic valves, warranting the need for reintervention, as well as bleeding risks associated with lifelong anticoagulation with mechanical valves.^22^ Our study found increasing adoption of TAVR in patients aged 50-64 years requiring AVR compared with SAVR, which aligns with previous retrospective studies.^23^^,^^24^ The temporal increase in TAVR among patients aged 50-64 years may be attributed to the following: (1) improved safety and efficacy outcomes with TAVR due to advancements in procedural techniques and transcatheter devices^6^; (2) patient preference for a less-invasive AVR method; (3) feasibility of TAVR across a wide range of anatomical variances, along with the expansion of patient eligibility to include intermediate surgical risk patients in 2016 and low surgical risk patients in 2019^2^, ^3^, ^4^, ^5^, ^6^; and (4) increased availability of TAVR at hospitals across the United States.^25^

Among patients aged 50-64 years, TAVR was associated with lower odds of in-hospital mortality, stroke, AKI, major bleeding, and blood transfusion compared with SAVR. These findings are congruent with previous studies,^26^^,^^27^ including a retrospective study by Gad et al,^26^ which found lower rates of AKI and cerebrovascular accidents among patients <60 years old who underwent TAVR compared with those of SAVR,^26^ as well as a meta-analysis by Koshy et al,^27^ which found significantly lower mortality, major bleeding, and AKI among low-risk patients who underwent TAVR compared with those among patients who underwent SAVR.^27^ Lower odds of in-hospital mortality and procedural complications among TAVR-treated patients aged 50-64 years may be explained by the minimally invasive nature of the procedure, experienced operators, well-designed TAVR devices with compact sheath sizes, and the use of conscious sedation.^27^

Vascular complications were higher among patients aged 50-64 years who underwent TAVR compared with SAVR. This is consistent with previous reports,^28^^,^^29^ including a meta-analysis by Fu et al,^29^ which found increased vascular complications among low-risk and intermediate-risk patients who underwent TAVR compared with those among patients who underwent SAVR.^29^ Although our study cohort encompassed patients aged 50-64 years, vascular complications appear to be prominent after TAVR, irrespective of age, as highlighted in a retrospective study by Oh et al,^30^ which noted vascular complications after TAVR were similar between patients aged 65-79 years and patients aged ≥80 years.^30^ Female sex, distorted vascular anatomy, arterial access location and technique, sheath size, and anticoagulation use are factors known to contribute to vascular complications following TAVR.^31^ The impact of vascular complications on adverse outcomes among young patients undergoing TAVR remains a significant concern, with previous studies noting longer LOS, higher hospitalization costs, decreased quality of life, increased mortality, and greater risks of developing both infections and bleeding complications among those affected.^31^ While our study noted higher vascular complications among patients aged 50-64 years undergoing TAVR vs SAVR, vascular complications may decline in the coming years due to smaller TAVR delivery sheaths, enhanced vascular closure instruments, optimized patient selection criteria, and a continued rise in high-volume, experienced TAVR centers.^31^

Our study found that TAVR was associated with shorter hospital LOS and lower nonhome discharges compared with SAVR in patients aged 50-64 years. These findings are similar to previous studies comparing LOS and disposition outcomes between TAVR and SAVR.^32^^,^^33^ Shorter LOS and lower nonhome discharges among patients aged 50-64 years who underwent TAVR are likely multifactorial and include the following: (1) the use of conscious sedation allowing for quicker recovery periods and decreased periprocedural complications^32^; (2) fewer stroke, AKI, and bleeding complications with TAVR than those with SAVR; and (3) provider practices in selecting patients who would be candidates for early discharge after TAVR.^32^

Among patients aged 50-64 years, 90-day all-cause and stroke readmissions and 180-day all-cause, heart failure, and stroke readmissions were higher with TAVR than those with SAVR. Although previous studies found similar readmissions between TAVR and SAVR, patients aged <65 years were excluded from those studies.^34^ Potential factors for the higher readmission rates with TAVR than those for SAVR include the following: (1) higher rates of heart block, PPM placement, and vascular complications following TAVR; (2) higher nonhome discharges in the SAVR group; (3) higher incidence of paravalvular leaks reported with TAVR previously, which has been associated with a higher risk of rehospitalization, even if mild^35^^,^^36^; (4) differences in follow-up practices and access to postprocedural care between TAVR-treated and SAVR-treated patients; (5) sicker TAVR-treated patient population despite propensity score matching, as those who underwent TAVR may have been at high surgical risk for SAVR, in accordance with current guideline recommendations favoring SAVR for patients <65 years old^1^; and (6) potential confounding from out-of-hospital deaths, which are not captured in the NRD. Further studies evaluating long-term readmissions following TAVR vs SAVR in patients aged 50-64 years are warranted.

Transcatheter aortic valve replacement with a bioprosthetic valve in patients aged 50-64 years highlights the importance of lifetime management while taking into account patient-specific characteristics and patient wishes: if structural valve deterioration occurs after 10-15 years, even if valve-in-valve TAVR is performed at that point, these patients are at risk to outlive 2 TAVR valves. Future long-term data on TAVR valve durability will be critical to determining the appropriateness of TAVR in patients aged 50-64 years. Additionally, long-term durability data for TAVR in SAVR, as well as TAVR in TAVR are needed to help determine the ideal sequence of valve intervention from index AVR.

This study has several important limitations to acknowledge. First, in a retrospective NRD study using administrative claims codes, incorrect coding could lead to inaccurate data. Second, the retrospective nature of the study makes it subject to inherent selection bias, and randomized controlled trials are needed to confirm the present findings. Third, despite propensity score matching resulting in similar measurable baseline characteristics between the 2 study cohorts, there likely remain unmeasured confounders that may affect the findings of this study. Fourth, detailed baseline and procedural characteristics, such as echocardiographic and computed tomographic data, access site, valve type (eg, balloon-expandable or self-expanding; mechanical; or bioprosthetic), valve size, and periprocedural medications, are unavailable in the NRD, which can lead to unmeasured bias. Fifth, although an ICD-10 code exists for BAV, we were unable to accurately assess the prevalence of BAV due to the absence of echocardiographic data in the NRD. Sixth, validated risk scores such as the Society of Thoracic Surgeons score and the Hospital Frailty Risk Score are not captured by the NRD, limiting patient risk assessment. Seventh, during the study period (2016-2021), TAVR was approved for patients at high surgical risk (since 2012) and intermediate-risk (since 2016), with approval for low-risk patients only occurring in 2019. As a result, most TAVR-treated patients in our study were likely at intermediate or high surgical risk, and the present findings may not be generalizable to low-risk patients aged 50-64 years. Eighth, given the lack of data on out-of-hospital deaths, patients who died at home within 180 days were counted as patients without a readmission within 180 days. Finally, our study was limited to in-hospital outcomes and 180-day readmissions. Studies exploring the long-term outcomes of TAVR vs SAVR in patients aged 50-64 years are still needed using newer databases such as TriNetX and Epic Cosmos.

Despite these limitations, this study adds meaningfully to the literature by describing the relative use and comparative outcomes of TAVR vs SAVR in patients aged 50-64 years. The NRD is well validated for outcomes studies like this one and undergoes serial data accuracy checks and quality control. In addition, NRD data are geographically diverse, including a nationally representative sample of centers and operators, and hence reliably reflect real-world practice and outcomes.

## Conclusion

This observational analysis of a large national database demonstrated that TAVR is increasingly performed among patients aged 50-64 years and is offered to patients with more comorbidities instead of SAVR. Compared with SAVR, TAVR was associated with lower in-hospital mortality and resource utilization but higher readmissions. Randomized controlled trials exploring the long-term comparative safety and effectiveness of TAVR vs SAVR among patients aged 50-64 years are warranted to confirm these observational findings.
