# Supplementary material for: Transcatheter Versus Surgical Aortic Valve Replacement in Patients Aged 50 to 64 Years in the United States
Source: J Soc Cardiovasc Angiogr Interv. 2025 Oct 21;4(11):103991. doi: 10.1016/j.jscai.2025.103991 (PMC12664615; doi:10.1016/j.jscai.2025.103991)

**Supplementary Material**

**Table S1.** Summary of NRD states, hospitals, and inpatient stays (adopted from <https://hcup-us.ahrq.gov/db/nation/nrd/NRDIntroduction2021.pdf>)

| **Year** | **States** | **Number of States for Discharges (Age ≥1 Year)** | **Number of Hospitals** | **Number of Discharges in the NRD** | |
| --- | --- | --- | --- | --- | --- |
|  |  |  |  | **Unweighted** | **Weighted** |
| 2021 | AK, AR, CA, CO, DE, FL, GA, HI, IA, IN, LA, MA, MD, ME, MO, MS, NE, NM, NY, OR, PA, SC, SD, TN, UT, VA, VT, WA, WI, WY | 30 | 2,505 | 16,805,508 | 33,420,845 |
| 2020 | AK, AR, CA, CO, DE, FL, GA, HI, IA, IN, LA, MA, MD, ME, MO, MS, NE, NM, NV, NY, OR, PA, SC, SD, TN, UT, VA, VT, WA, WI, WY | 31 | 2,539 | 16,692,694 | 32,336,475 |
| 2019 | AK, AR, CA, CO, DE, FL, GA, HI, IA, IN, LA, MA, MD, MO, MS, NE, NM, NV, NY, OR, PA, SC, SD, TN, UT, VA, VT, WA, WI, WY | 30 | 2,507 | 18,132,856 | 35,399,480 |
| 2018 | AK, AR, CA, DE, FL, GA, IA, IN, LA, MA, MD, MO, MS, NE, NM, NV, NY, OR, PA, SC, SD, TN, UT, VA, VT, WA, WI, WY | 28 | 2,430 | 17,686,511 | 35,460,557 |
| 2017 | AK, AR, CA, DE, FL, GA, IA, IN, LA, MA, MD, MO, MS, NE, NM, NV, NY, OR, PA, SC, SD, TN, UT, VA, VT, WA, WI, WY | 28 | 2,454 | 17,978,754 | 35,790,513 |
| 2016 | AK, AR, CA, FL, GA, HI, IA, LA, MA, MD, MO, MS, NE, NM, NV, NY, OR, PA, SC, SD, TN, UT, VA, VT, WA, WI, WY | 27 | 2,355 | 17,197,683 | 35,660,906 |

# Table S2. Strengthening the Reporting of Observational Studies in Epidemiology (STROBE) guidelines to report the study findings

STROBE Statement—Checklist of items that should be included in reports of ***cross-sectional studies***

|  | **Item No** | **Recommendation** | **Page No** |
| --- | --- | --- | --- |
| **Title and abstract** | 1 | (*a*) Indicate the study’s design with a commonly used term in the title or the abstract | 3 |
|  |  | (*b*) Provide in the abstract an informative and balanced summary of what was done and what was found | 3 |
| **Introduction** | | | |
| Background/rationale | 2 | Explain the scientific background and rationale for the investigation being reported | 6 |
| Objectives | 3 | State specific objectives, including any prespecified hypotheses | 6,7 |
| **Methods** | | | |
| Study design | 4 | Present key elements of study design early in the paper | 7,8 |
| Setting | 5 | Describe the setting, locations, and relevant dates, including periods of recruitment, exposure, follow-up, and data collection | 7,8 |
| Participants | 6 | (*a*) Give the eligibility criteria, and the sources and methods of selection of participants | 7,8 |
| Variables | 7 | Clearly define all outcomes, exposures, predictors, potential confounders, and effect modifiers. Give diagnostic criteria, if applicable | 8,9 |
| Data sources/ measurement | 8* | For each variable of interest, give sources of data and details of methods of assessment (measurement). Describe comparability of assessment methods if there is more than one group | 6-8 |
| Bias | 9 | Describe any efforts to address potential sources of bias | 10 |
| Study size | 10 | Explain how the study size was arrived at | 7-9 |
| Quantitative variables | 11 | Explain how quantitative variables were handled in the analyses. If applicable, describe which groupings were chosen and why | 7-9 |
| Statistical methods | 12 | (*a*) Describe all statistical methods, including those used to control for confounding | 9-11 |
|  |  | (*b*) Describe any methods used to examine subgroups and interactions | N/A |
|  |  | (*c*) Explain how missing data were addressed | 10 |
|  |  | (*d*) If applicable, describe analytical methods taking account of sampling strategy | 10,11 |
|  |  | (*e*) Describe any sensitivity analyses | N/A |
| **Results** | | | |
| Participants | 13* | (a) Report numbers of individuals at each stage of study—eg numbers potentially eligible, examined for eligibility, confirmed eligible, included in the study, completing follow-up, and analysed | 11 |
|  |  | (b) Give reasons for non-participation at each stage | 11 |
|  |  | (c) Consider use of a flow diagram | 11 |
| Descriptive data | 14* | (a) Give characteristics of study participants (eg demographic, clinical, social) and information on exposures and potential confounders | 11 |
|  |  | (b) Indicate number of participants with missing data for each variable of interest | 10 |
| Outcome data | 15* | Report numbers of outcome events or summary measures | 12,13 |
| Main results | 16 | (*a*) Give unadjusted estimates and, if applicable, confounder-adjusted estimates and their precision (eg, 95% confidence interval). Make clear which confounders were adjusted for and why they were included | 12,13 |
|  |  | (*b*) Report category boundaries when continuous variables were categorized | N/A |
|  |  | (*c*) If relevant, consider translating estimates of relative risk into absolute risk for a meaningful time period | N/A |
| Other analyses | 17 | Report other analyses done—eg analyses of subgroups and interactions, and sensitivity analyses | N/A |
| **Discussion** | | | |
| Key results | 18 | Summarise key results with reference to study objectives | 13 |
| Limitations | 19 | Discuss limitations of the study, taking into account sources of potential bias or imprecision. Discuss both direction and magnitude of any potential bias | 16,17 |
| Interpretation | 20 | Give a cautious overall interpretation of results considering objectives, limitations, multiplicity of analyses, results from similar studies, and other relevant evidence | 13-17 |
| Generalisability | 21 | Discuss the generalisability (external validity) of the study results | 16,17 |
| **Other information** | | | |
| Funding | 22 | Give the source of funding and the role of the funders for the present study and, if applicable, for the original study on which the present article is based | 1 |

*Give information separately for exposed and unexposed groups.

**Note:** An Explanation and Elaboration article discusses each checklist item and gives methodological background and published examples of transparent reporting. The STROBE checklist is best used in conjunction with this article (freely available on the Web sites of PLoS Medicine at http://www.plosmedicine.org/, Annals of Internal Medicine at http://www.annals.org/, and Epidemiology at http://www.epidem.com/). Information on the STROBE Initiative is available at www.strobe-statement.org.

**Table S3.** ICD-10 diagnosis (CM) and procedure (PCS) codes

| **Inclusion** | **ICD-10 PCS codes** |
| --- | --- |
| TAVR | 02RF37H, 02RF38H, 02RF3JH, 02RF3KH, X2RF332, 02RF37Z, 02RF38Z, 02RF3JZ, 02RF3KZ |
| SAVR | 02RF07Z, 02RF08Z, 02RF0JZ, 02RF0KZ, X2RF032 |

| **Exclusion** | **ICD-10 CM/PCS codes** |
| --- | --- |
| PCI | 02C03ZZ, 02C04ZZ, 02C13ZZ, 02C14ZZ, 02C23ZZ, 02C24ZZ, 02C33ZZ, 02C34ZZ, 02703ZZ, 02704ZZ, 02713ZZ, 02714ZZ, 02723ZZ, 02724ZZ, 02733ZZ, 02734ZZ |
| CABG | 0210093, 0210098, 0210099, 021009C, 021009F, 021009W, 02100A3, 02100A8, 02100A9, 02100AC, 02100AF, 02100AW, 02100J3, 02100J8, 02100J9, 02100JC, 02100JF, 02100JW, 02100K3, 02100K8, 02100K9, 02100KC, 02100KF, 02100KW, 02100Z3, 02100Z8, 02100Z9, 02100ZC, 02100ZF, 0211093, 0211098, 0211099, 021109C, 021109F, 021109W, 02110A3, 02110A8, 02110A9, 02110AC, 02110AF, 02110AW, 02110J3, 02110J8, 02110J9, 02110JC, 02110JF, 02110JW, 02110K3, 02110K8, 02110K9, 02110KC, 02110KF, 02110KW, 02110Z3, 02110Z8, 02110Z9, 02110ZC, 02110ZF, 0212093, 0212098, 0212099, 021209C, 021209F, 021209W, 02120A3, 02120A8, 02120A9, 02120AC, 02120AF, 02120AW, 02120J3, 02120J8, 02120J9, 02120JC, 02120JF, 02120JW, 02120K3, 02120K8, 02120K9, 02120KC, 02120KF, 02120KW, 02120Z3, 02120Z8, 02120Z9, 02120ZC, 02120ZF, 0213093, 0213098, 0213099, 021309C, 021309F, 021309W, 02130A3, 02130A8, 02130A9, 02130AC, 02130AF, 02130AW, 02130J3, 02130J8, 02130J9, 02130JC, 02130JF, 02130JW, 02130K3, 02130K8, 02130K9, 02130KC, 02130KF, 02130KW, 02130Z3, 02130Z8, 02130Z9, 02130ZC, 02130ZF |
| Mitral valve surgery | 02RG07Z, 02RG08Z, 02RG0JZ, 02RG0KZ, 02QG0ZE, 02QG0ZZ, 02UG07E, 02UG07Z, 02UG08E, 02UG08Z, 02UG0JE, 02UG0JZ, 02UG0KE, 02UG0KZ |
| Tricuspid valve surgery | 02UJ0KZ, 027J04Z, 02NJ0ZZ, 02QJ0ZZ, 02UJ07Z, 02UJ08Z, 02UJ0JZ, 027J0DZ, 027J0ZZ, 02BJ0ZX, 02BJ0ZZ, 02RJ07Z, 02RJ0JZ, 02CJ0ZZ, 027J04Z, 02RJ08Z, 02RJ0KZ, 02WJ08Z, 02WJ0JZ, 02WJ07Z, 02WJ0KZ |
| Pulmonic valve surgery | 02WH0KZ, 02WH07Z, 02WH0JZ, 027H04Z, 02RH08Z, 02TH0ZZ, 02RH0JZ, 02RH07Z, 02BH0ZZ, 02BH0ZX, 027H0ZZ, 027H0DZ, 025H0ZZ, 02UH0KZ, 02UH0JZ, 02UH08Z, 02UH07Z, 02QH0ZZ, 02NH0ZZ |
| Other CV surgery | 02QX0ZZ, 02QW0ZZ, 02QV0ZZ, 02QT0ZZ, 02QS0ZZ, 02QR0ZZ, 02QQ0ZZ, 02QP0ZZ, 02QN0ZZ, 02QM0ZZ, 02QL0ZZ, 02QK0ZZ, 02QD0ZZ, 02QC0ZZ, 02QB0ZZ, 02QA0ZZ, 02Q90ZZ, 02Q80ZZ, 02Q70ZZ, 02Q60ZZ, 02Q50ZZ, 02Q40ZZ, 02RX07Z, 02RX08Z, 02RX0JZ, 02RX0KZ, 02RW07Z, 02RW08Z, 02RW0JZ, 02RW0KZ, 02RV07Z, 02RV08Z, 02RV0JZ, 02RV0KZ, 02RT07Z, 02RT08Z, 02RT0JZ, 02RT0KZ, 02RS07Z, 02RS08Z, 02RS0JZ, 02RS0KZ, 02RR07Z, 02RR08Z, 02RR0JZ, 02RR0KZ, 02RQ07Z, 02RQ08Z, 02RQ0JZ, 02RQ0KZ, 02RP07Z, 02RP08Z, 02RP0JZ, 02RP0KZ, 02RN07Z, 02RN08Z, 02RN0JZ, 02RN0KZ, 02RM07Z, 02RM08Z, 02RM0JZ, 02RM0KZ, 02RL07Z, 02RL08Z, 02RL0JZ, 02RL0KZ, 02RK07Z, 02RK08Z, 02RK0JZ, 02RK0KZ, 02RD07Z, 02RD08Z, 02RD0JZ, 02RD0KZ, 02R907Z, 02R908Z, 02R90JZ, 02R90KZ, 02R707Z, 02R708Z, 02R70JZ, 02R70KZ, 02R607Z, 02R608Z, 02R60JZ, 02R60KZ, 02R507Z, 02R508Z, 02R50JZ, 02R50KZ |
| Infective endocarditis | I33.0 |
| Prosthetic valve dysfunction | T8201XA, T8202XA, T8203XA, T8209XA, T82221A, T82222A, T82223A, T82228A |

| **Baseline characteristics** | **ICD-10 CM codes** |
| --- | --- |
| Bicuspid aortic valve | Q23.1, Q23.83 |
| Diabetes mellitus | E10.0, E10.1, E10.9, E11.0, E11.1, E11.9, E12.0, E12.1, E12.9, E13.0, E13.1, E13.9, E14.0, E14.1, E14.9, E10.2-E10.8, E11.2-E11.8, E12.2-E12.8, E13.2-E13.8, E14.2-E14.8 |
| Hypertension | I10.x, I11.x-I13.x, I15.x |
| Dyslipidemia | E78.x |
| Nicotine/tobacco use | F17.x, Z72.0, Z87.891 |
| Alcohol abuse | F10, E52, G62.1, I42.6, K29.2, K70.0, K70.3, K70.9, T51.x, Z50.2, Z71.4, Z72.1 |
| Drug abuse | F11.x-F16.x, F18.x, F19.x, Z71.5. Z72.2 |
| Obesity | E66.x |
| Coronary artery disease | I25.x |
| Peripheral vascular disease | I70.x, I71.x, I73.1, I73.8, I73.9, I77.1, I79.0, I79.2, K55.1, K55.8, K55.9, Z95.8, Z95.9 |
| Atrial fibrillation/flutter | I48.x |
| Congestive heart failure | I09.9, I11.0, I13.0, I13.2, I25.5, I42.0, 142.5-I42.9, I43.x, I50.x, P29.0 |
| Renal failure | I12.0, I13.1, N18.x, N19.x, N25.0, Z49.0-Z49.2, Z94.0, Z199.2 |
| Dialysis dependent | Z99.2 |
| Liver disease | B18.x, I85.x, I86.4, I98.2, K70.x, K71.1, K71.3-K71.5, K71.7, K72.x-K74.x, K76.0, K76.2-K76.9. Z94.4 |
| Chronic pulmonary disease | I27.8, 127.9, J40.x-J47.x, J60.x-J67.x, J68.4, J70.1, J70.3 |
| Obstructive sleep apnea | G47.33 |
| Coagulopathy | D65-D68.x, D69.1, D69.3-D69.6 |
| Cancer | C0x.x, C1x.x, C2x.x, C30.x, C31.x, C32.x, C33.x, C34.x, C37.x, C38.x, C39.x, C40.x, C41.x, C43.x, C45.x, C46.x, C47.x, C48.x, C49.x, C50, C51-58.x, C60-63.x, C76.x, C80.1, C81.x, C82.x, C83.x, C84.x, C85.x, C88.x, C9x.x |
| Malnutrition | E43, E44.x, E45, E46 |
| Dementia | F01.x, F02.x, F03.x, F04, F05, F06.1, F06.8, G13.2, G13.8, G30.x, G31.0x, G31.1, G31.2, G91.4, G94, R41.81, R54 |
| Depression | F20.4, F31.3-F31.5, F32.x, F33.x, F34.1, F41.2, F43.2 |
| **Previous history** | |
| Myocardial infarction | I25.2 |
| Stroke/TIA | Z86.73 |
| Cardiac arrest | Z86.74 |
| PCI | Z98.61, Z95.5 |
| CABG | Z95.1 |
| ICD | Z95.810 |
| PPM | Z95.0 |

| **In-hospital outcomes** | **ICD-10 CM/PCS codes** |
| --- | --- |
| Stroke | I60.x, I61.x, I62.x, I63.x, I67.81, I67.82, G45.x, G46.x, H34.0x, H34.1x, H34.2x, I97.820, I97.810 |
| Heart block | I44.x, I45.0, I45.1x, I45.2, I45.3, I45.4, I45.5 |
| PPM placement | 0JH634Z, 0JH635Z, 0JH636Z, 0JH63PZ, 0JH834Z, 0JH835Z, 0JH836Z, 0JH83PZ, 0JH604Z, 0JH605Z, 0JH606Z, 0JH60PZ, 0JH804Z, 0JH805Z, 0JH806Z, 0JH80PZ |
| Acute kidney injury | N17.x, N99.0 |
| Major bleeding | I97.610, I97.618, I97.620, I97.410, I97.418, I97.42, T82.837 |
| Blood transfusion | 30233H0, 30233H1, 30233N0, 30233N1, 30233P0, 30233P1, 30230H0, 30230H1, 30230N0, 30230N1, 30230P0, 30230P1, 30243H0, 30243H1, 30243N0, 30243N1, 30243P0, 30243P1, 30240H0, 30240H1, 30240N0, 30240N1, 30240P0, 30240P1 |
| Vascular complications | I77.0, I72.x, L76.32, L76.02, I97.630, I97.638, K66.1, I26.92, I26.93, I26.94, I26.99, I26.02, I26.09, I82.4x, T81.718A, T81.719A |

**Table S4.** Variables used in the propensity-score matching model to compute adjusted in-hospital outcomes and readmissions

| **Demographic characteristics** |
| --- |
| Age |
| Biological sex |
| Primary payer |
| Income quartile |
| **Hospital characteristics** |
| Location/teaching status |
| Bed size |
| Elective admission |
| Weekend admission |
| **Comorbidities** |
| Bicuspid aortic valve |
| Elixhauser comorbidity index |
| Charlson comorbidity index |
| Diabetes mellitus |
| Hypertension |
| Dyslipidemia |
| Nicotine/tobacco use |
| Alcohol abuse |
| Drug abuse |
| Obesity |
| Coronary artery disease |
| Peripheral vascular disease |
| Atrial fibrillation/atrial flutter |
| Congestive heart failure |
| Renal failure |
| Dialysis dependent |
| Liver disease |
| Chronic pulmonary disease |
| Obstructive sleep apnea |
| Coagulopathy |
| Cancer |
| Malnutrition |
| Dementia |
| Depression |
| **Previous** **history** |
| Myocardial infarction |
| Stroke/TIA |
| Cardiac arrest |
| PCI |
| CABG |
| ICD |
| PPM |

**Figure S1.** Absolute standardized mean differences for variables included in the propensity-score matching model for patients aged 50-64 years undergoing TAVR vs. SAVR

**
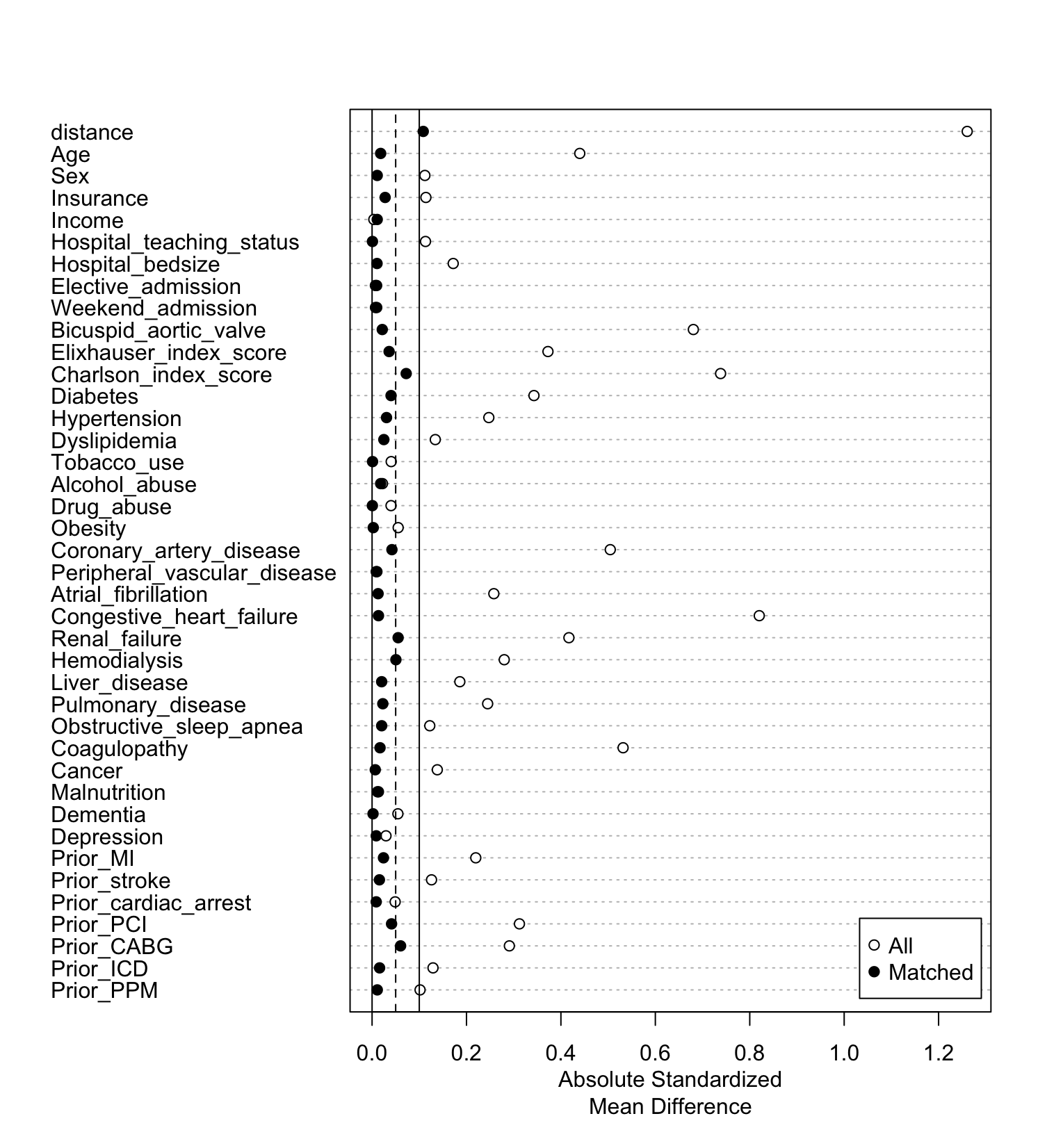
**

**Figure S2.** Distribution of propensity scores for patients aged 50-64 years undergoing TAVR vs. SAVR


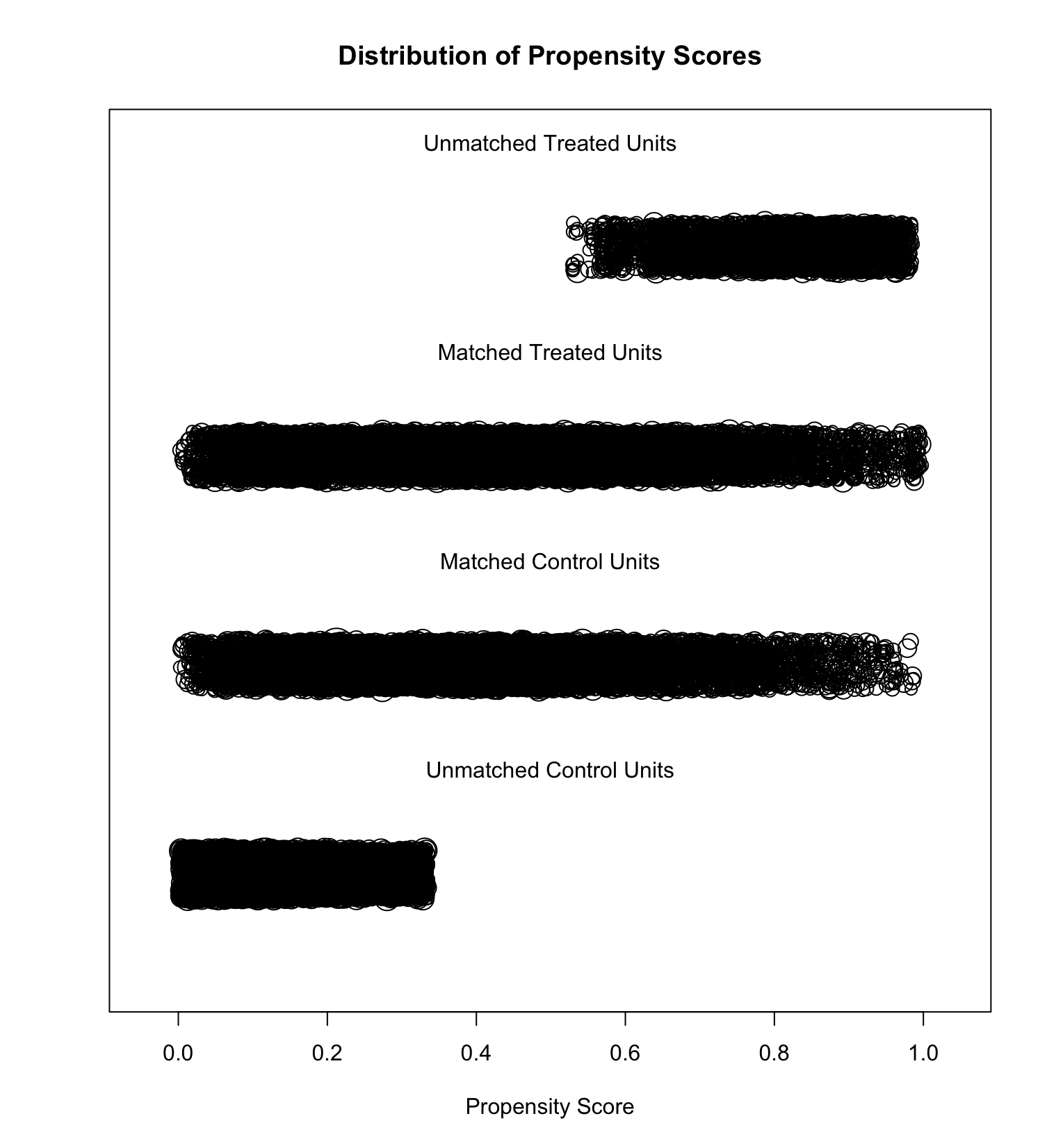


**Figure S3.** Propensity scores among patients aged 50-64 years undergoing TAVR vs. SAVR before and after matching


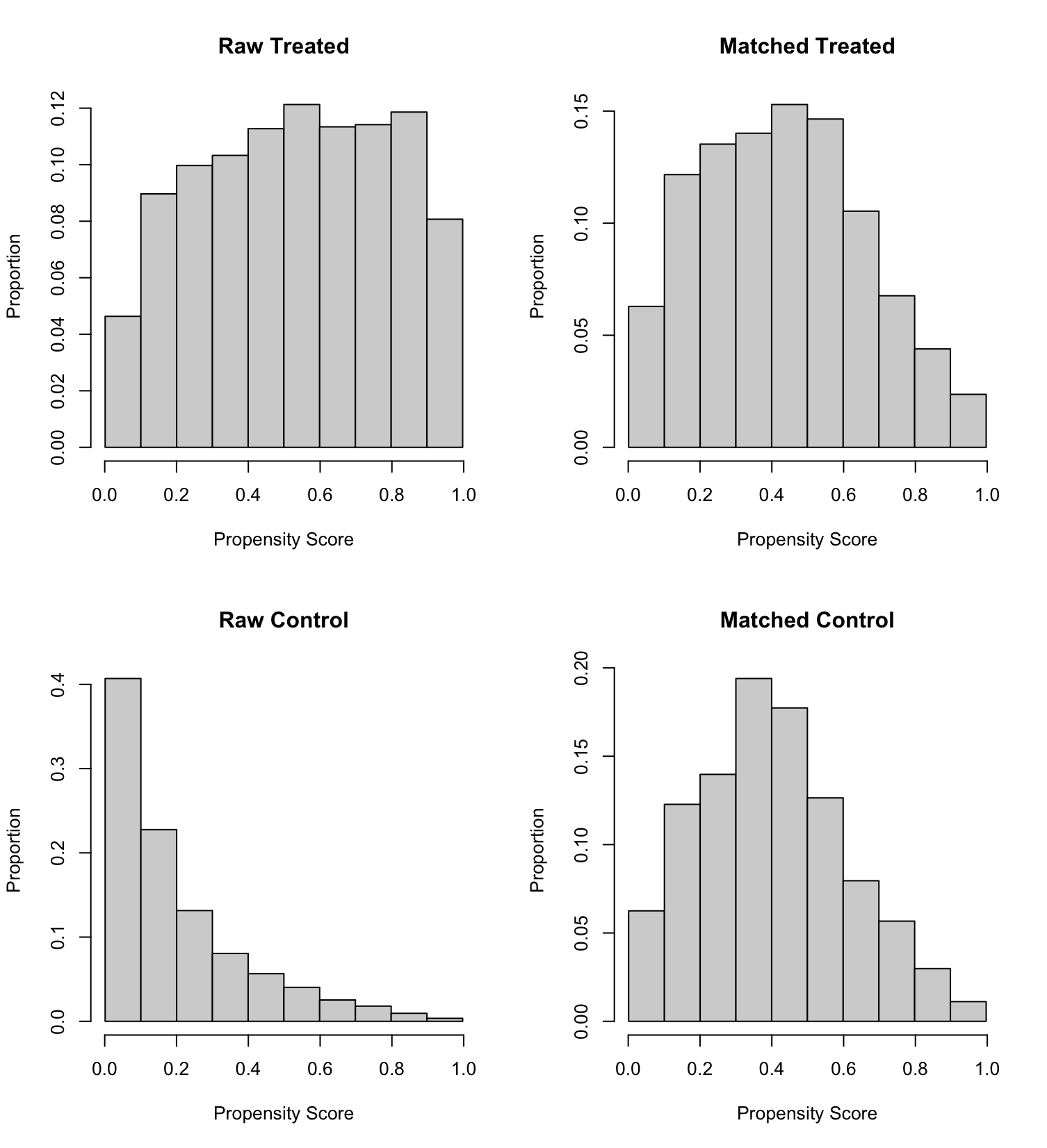


**Figure S4.** Distributional balance for “distance” among patients aged 50-64 years undergoing TAVR vs. SAVR before and after matching


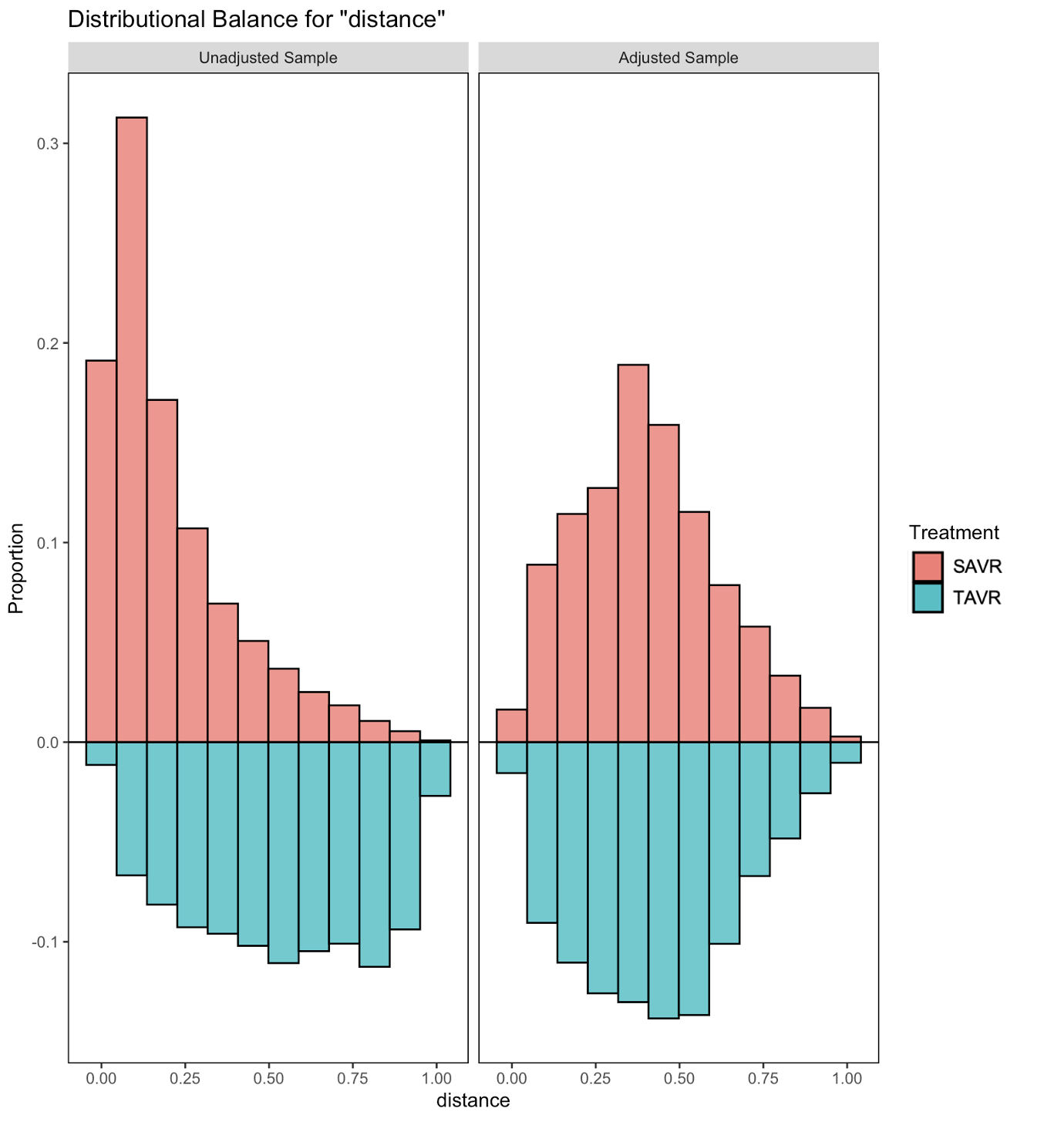

Supplement: Supplementary Material [file mmc1.docx]
